# A Hybrid Handover Scheme for Vehicular VLC/RF Communication Networks

**DOI:** 10.3390/s24134323

**Published:** 2024-07-03

**Authors:** Linqiong Jia, Shicheng Feng, Yijin Zhang, Jin-Yuan Wang

**Affiliations:** 1 The School of Electronic and Optical Engineering, Nanjing University of Science and Technology, Nanjing 210094, China; shichengfengf1102@njust.edu.cn; 2 The School of Communication and Information Engineering, Nanjing University of Posts and Telecommunications, Nanjing 210003, China; jywang@njupt.edu.cn

**Keywords:** vehicular VLC/RF communication networks, vertical handover scheme, horizontal handover, Markov decision process (MDP), value iteration algorithm

## Abstract

Visible light communication (VLC) is a promising complementary technology to its radio frequency (RF) counterpart to satisfy the high quality-of-service (QoS) requirements of intelligent vehicular communications by reusing LED street lights. In this paper, a hybrid handover scheme for vehicular VLC/RF communication networks is proposed to balance QoS and handover costs by considering the vertical handover and horizontal handover together judging from the mobile state of the vehicle. A Markov decision process (MDP) is formulated to describe this hybrid handover problem, with a cost function balancing the handover consumption, delay, and reliability. A value iteration algorithm was applied to solve the optimal handover policy. The simulation results demonstrated the performance of the proposed hybrid handover scheme in comparison to other benchmark schemes.

## 1. Introduction

The ongoing increased development of intelligent transportation systems and autonomous vehicles makes the connection between the road and vehicles more relevant, which greatly improves the quality-of-service (QoS) requirements for vehicular communications, so as to increase road safety and intelligence. The radio frequency (RF) spectrum resources for wireless communication have become saturated, thus RF communication can hardly keep up with the vehicular communication’s demand for a huge amount of data, high data rate, and high mobility. Visible light communication (VLC) is considered a candidate solution to enhance the performance of the system and overcome bandwidth limitations [1]. The wide use of light emitting diodes (LED) in cars and street lights also encourages the exploitation of the VLC paradigm in vehicular applications [2].

VLC is highly dependent on line of sight (LoS) links, which cannot always be guaranteed. The random movement of vehicles and weather conditions are potential factors causing transmission interruptions in outdoor environments. Therefore, VLC alone cannot provide continuous data access. Integrated VLC/RF networks are receiving increasing attention, due to their improved transmission robustness [3].

Handover management has been proposed to improve the efficiency of resource utilization and the QoS in VLC networks and heterogeneous VLC/RF networks [1]. The process of mobile terminals (MTs) switching communication session to another access point (AP) in the same network is called horizontal handover (HHO), which is mainly required due to mobility [2]. When link interruptions or severe performance degradation occur, the MT’s ongoing connection will be switched to another network in heterogeneous systems with a guaranteed QoS, which is usually referred to as vertical handover (VHO) [3].

Several HHO schemes have been proposed to enhance the QoS of VLC networks. The performance of a soft handover scheme using a received signal strength indicator (RSSI) as a metric was evaluated in [4]. For properly supporting a high level of mobility, an HHO technique was studied based on the interference-to-noise ratio and interference-to-interference ratio in vehicular visible light networks [2]. A distance-based HHO procedure was proposed for a vehicle-to-infrastructure (V2I) VLC system, to maximize the signal quality subject to a predetermined missing handover rate constraint [5]. A novel architecture using a multipath-transmission control protocol was optimized to improve the handover performance in terms of network outage duration in vehicular VLC networks [6]. A dynamic soft handover algorithm based on coordinated multipoint (CoMP) transmission was investigated in [7,8], which could maintain a stable signal quality regardless of vehicle velocity. A coordinated scheduling solution with a soft handover technique was offered to increase the achievable throughput and link reliability for VLC networks in [1]. Moreover, ref. [9] presented a two-tier LiFi network and analyzed the closed-form expressions of the received optical signal intensity, time-to-trigger, and user mobility for the cross-tier handover rate between primary and secondary cells.

There have been several works investigating the VHO strategy for heterogeneous VLC/RF networks, because VHO is a process that can deal with the interruption of VLC links [10]. An MDP-based VHO scheme was investigated to balance the switching costs and the delay requirements of indoor VLC-RF systems in [11]. Considering load balancing, a two-way selection handover scheme was proposed to improve the overall system performance [12]. A fuzzy-logic (FL)-based decision-making VHO algorithm was proposed for radio frequency and optical wireless (RF/OW) systems, which was capable of providing better QoS to users in terms of packet transfer delay [3]. A load balancing scheme aiming at improving the user QoS was realized by soft handover in hybrid VLC/WiFi networks [13]. A flexible and holistic self-optimizing algorithm was proposed for highly mobile scenarios, which was able to control the handover parameters of all VLC APs under the coverage of the LTE eNodeB based on the Q-learning approach in [14]. The VHO threshold was derived based on an exact mathematical modeling of a vehicular VLC platoon in [15].

Among the above vertical handover solutions, the MDP-based method has been widely used in the handover strategies of heterogeneous networks [16,17,18,19,20]. A new efficient handover decision algorithm based on an MDP was proposed to optimize the overall service experience of users in millimeter wave heterogeneous networks in 5G cellular systems in [16]. Ref. [17] presented a novel energy-efficient and delay-aware handover decision policy based on an MDP for a macro-femto cell wireless network. For hybrid WiFi and infrared uplink transmission, a link switching scheme was presented based on an MDP, with the objective of minimizing the transfer delay of data [19]. An MDP model was proposed to manage user mobility and handovers among the cells in heterogeneous ultra-dense networks in 5G cellular systems, with the Stackelberg competition model introduced to increase the relay node selected and to guarantee communication quality and load balancing [20].

The deep reinforcement learning algorithm, as an advanced MDP-based model, has also been applied to cope with the handover problems of neural networks trained to replace unknown state transition rates or reward functions [21,22,23,24]. An adaptive handover mechanism, including a seamless handover protocol and a selection algorithm, was optimized by a deep reinforcement learning (DRL) method in VLC/6G hybrid indoor networks with ultradense deployed VLC APs [21]. A multi-UAV cell association and their moving velocity decision problem in multi-UAV networks was formulated as an MDP, and a deep reinforcement learning algorithm was developed to improve both the transportation and communication performance with the branching dueling Q-network (BDQ) and branching dueling double deep Q-network (Dueling DDQN) [22]. A complex inter-satellite beam handover that required balanced channel capacity was characterized as an MDP and solved using an improved deep reinforcement learning (DRL) algorithm to determine the handover satellite and handover beam [23].

In heterogeneous VLC/RF vehicular networks, persistent transmission via a VLC LoS link is always preferable, due to its better perceptible QoS. However, MTs suffer from severe QoS degradation when the LoS link is interrupted. VHO operation offers MTs wireless access by switching to RF links with reduced transmission rate. Once the VLC LoS link has ben recovered, switching back to the VLC AP is preferred for the QoS reasons. Because the LoS blocking does not last long for a common case, switching back and forth can cause the ping-pong effect. As a result, waiting for recovery of the VLC LoS link may be a better choice [11]. For vehicular VLC/RF networks, switching horizontally to the next accessible VLC AP is even better, because HHO is meant to be executed as the traffic moves and thus can avoid the additional signaling cost and latency of VHO. Therefore, we present an efficient hybrid handover strategy that can make a choice between VHO, HHO, or waiting in the case of VLC link disruption. Our main contributions are summarized as follows:

- With due consideration of the mobility of the vehicles, we put forward a hybrid handover strategy that makes proper handover decisions from one of VHO, early HHO, or waiting, so as to maintain persistent wireless access and improve the transmission performance in vehicular VLC/RF communication networks.
- A Markov decision process (MDP) is formulated to describe the hybrid handover problem, with a cost function considering the handover consumption, delay, and the reliability. A value iteration algorithm was applied to solve the decision-making problem.
- Simulation results demonstrated the performance of the proposed hybrid scheme comparison to benchmarks.

The rest of this paper is organized as follows: In Section 2, the system model and the main assumptions for the vehicular VLC/RF communication networks are introduced and the hybrid handover decision problem is proposed. In Section 3, the hybrid handover problem is illustrated as an MDP and the value iteration algorithm is applied for the optimal handover decisions. Section 4 shows a performance comparison of the proposed optimal handover scheme and other benchmark handover schemes. Finally, Section 5 concludes this paper.

## 2. System Model

### 2.1. System Model and Main Assumptions

**Figure 1:**
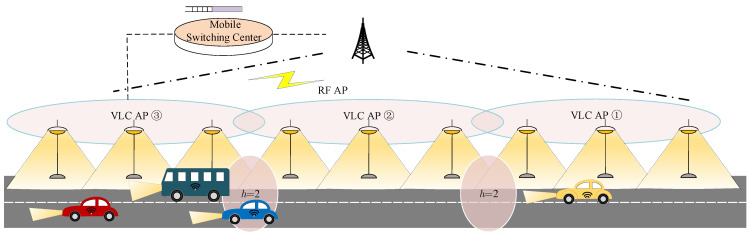
The vehicular VLC/RF communication networks.

The heterogeneous VLC/RF vehicular communication network provides vehicular mobile terminals with (VMTs) wireless access outdoors, as depicted in Figure 1. The RF APs are assumed to be the base stations of cellular networks, e.g., LTE eNodeB [14]. A VLC AP consists of several uniformly placed LED street lamps configured with communication modules [7]. Each VMT is equipped with at least one single photo-detector (PD) located at the top of the car, thus supporting both RF and VLC transmission in downlink vehicle-to-infrastructure (V2I) connections. As uplink traffic is always considerably less than downlink transmission, only RF transmission is employed in the uplinks [11].

To avoid inter-cell interference (ICI) for VLC APs, the available transmission spectrum is divided into two bands and consecutive VLC APs use different channel bands [4]. Since the visible light signal overlap only occurs between adjacent VLC APs, this bandplan strategy can eliminate ICI.

Let us consider a VTM connected to a VLC AP traveling along a given path. The packets are assumed to be transmitted through an LoS link. While driving, the VTM needs to perform HHO from the host VLC AP to the next AP (called the ’Target’ AP) [4]. When entering the overlapping area covered by two neighboring VLC APs (denoted by the pink circle labeled with h=2), the RSSI from the host AP $P_{H}$ reduces and the RSSI from the target AP $P_{T}$ increases. A conventional HHO process is triggered when $P_{T}>P_{H}+\mathrm{HOM}$, where HOM is called the handover margin [1]. The conventional HHO process can be triggered by LoS interruption of the VLC link, while fast recovery triggers switching back operation, which leads to ping-pong handovers consuming unnecessary signaling overheads [7]. Thus, the conventional HHO scheme was not adopted in the considered vehicular VLC/RF networks.

A soft HHO scheme is assumed to be applied for VLC HHO [4]. The VLC HHO process is triggered when $P_{T}>P_{R1}$, with $P_{R1}$ being an appropriate RSSI value for normal HHO executed while driving [4]. We also define an RSSI value $P_{R0}$ that is able to support VLC access as an early HHO threshold, thus $P_{R0}<P_{R1}$. The early HHO can be performed if VLC link blockage occurs on the condition of accessible target VLC APs. This threshold-based HHO scheme does not actually cause the ping-pong effect, because the original AP after HHO would not be identified as the target AP.

The RF link is assumed to be always available, because the coverage of an RF AP is much larger than that of a VLC AP and the RF signals can penetrate opaque obstruction and are less influenced by weather conditions. The mobile switching center is responsible for access selection and the handover process of the VMTs. The packets in the queue follow the first-in-first-out rule. Newly arrived packets are dropped when the buffer of the mobile switching center overflows.

### 2.2. Problem Motivation

In the considered vehicular VLC/RF networks, handover occurs due to mobility or the interruption and reconnection of VLC links. In the case of LoS VLC link interruption due to blockage from large vehicles or the influence of bad weather, the VTM can perform VHO or early HHO to maintain connectivity and improve reliability, or just wait for link recovery. If the VLC–LoS link is recovered after VHO execution, the switching-back operation will have an increased cost and delay. The early HHO can prevent the extra signaling costs and avoid severe network latency, since VTMs are supposed to be horizontally switched to the next VLC AP when moving. The waiting strategy for VLC link interruption results in increased delays and queue length, though it saves the cost of switching. This raises the handover decision-making problem of choosing an operation from one of VHO, early HHO, and waiting, and thus is called a ’hybrid’ handover scheme.

We aim to find an efficient hybrid handover scheme that makes a handover decision taking the switching cost and delay requirement into consideration. Here, it is assumed that both additional signaling cost and handover delay will be induced during VHO and HHO, but the signaling cost for HHO is set to 0 since will HHO take place sooner or later when the vehicle moves. This hybrid handover problem of vehicular VLC/RF heterogeneous networks can be formulated as an MDP and solved suing a dynamic programming algorithm, as shown in Section 3.

## 3. Hybrid Handover Scheme

In this section, the hybrid handover decision-making problem is formulated as a continuous-time MDP. The key components of the reinforcement learning are first defined, which include the state space, the action space, the transition probability, the cost function, and the policy [25]. Then, we reformulate the continuous-time MDP as a discrete problem through uniformization [26]. Lastly, a value iteration algorithm is applied to solve for the optimal policy, and the hybrid handover decision is made according to this policy.

### 3.1. The MDP Setup

The state space for the VTM access state in the vehicular VLC/RF communication networks is defined as follows:

(1) $$\Omega =\{\left(s,s^{\prime },h,b,w\right),s,s^{\prime }\in S,h\in H,b\in B,w\in W\}$$

where *s* and $s^{\prime }$ represent the availability of the host VLC AP and the next VLC AP, respectively. An ON–OFF model is adopted to represent the intermittent VLC links, where ON and OFF represent available VLC links and unavailable links, respectively. Therefore, S={On,Off} indicates the accessibility of VLC APs. The RF AP is assumed to always be available, thus not specified in the state space [14]. h∈H={1,2} is a location sign, with h=2 indicating the VMT located in the overlapping area of two adjacent VLC APs as shown in Figure 1 and 1 indicating the non-overlapping regions. b∈B={0,1,⋯,B} is the packet number in the queue, with *B* the network buffer size. w∈W={0,1} represents the transmission mode, where 0 means the VTM is connected to the RF AP and 1 is the VLC AP.

**Figure 2:**
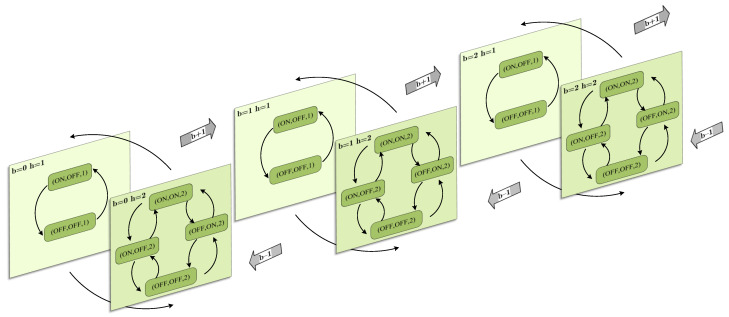
Illustration of states of the Markov process.

State transitions occur because of environmental variations, including packet arrival and departure, and changing optical channel. Figure 2 shows the MDP state transitions considering 6 states denoted as $\left(s,s^{\prime },h\right)$ for a certain queue length *b*. Obviously, the VTM is located in the overlapping areas of two adjacent VLC APs for states (On,On,2) and (Off,On,2).

The arrival process of the packets is modeled as a Poisson process with arriving rate λ (packets/s). The service processes of the RF APs and VLC APs are also Poisson, with service rate $\mu_{0}$ (packets/s) and $\mu_{1}$ (packets/s), respectively [27]. Let $\gamma_{1}$ ($s^{-1}$) and $\beta_{1}$ ($s^{-1}$) be the rates of the host VLC AP changing from available to unavailable and the inverse based on the ON–OFF model. Statistically, $1/\gamma_{1}$ and $1/\beta_{1}$ are the average durations of the host VLC AP staying ON and OFF, respectively. Similarly, $\gamma_{-1}$ ($s^{-1}$) and $\beta_{-1}$ ($s^{-1}$) represent the changing rate of the next VLC AP, from available to unavailable and the inverse. The availability of the host VLC AP and the next VLC AP is assumed to be independent.

A Poisson process with parameter $\alpha_{21}$ is used to describe the changes in the VTM from the VLC APs’ overlapping areas to the non-overlapping area. The reverse process is described by a Poisson process with parameter $\alpha_{12}$. These two changing rates depend on the vehicle velocity and the coverage area of the VLC APs. Since the overlapping area of VLC APs is commonly smaller than the non-overlapping area, $\alpha_{21}\geq \alpha_{12}$. The HHO execution process takes place in the overlapping area denoted by h=2. Normal HHO is assumed to have been executed when the VTM drives into the non-overlapping area from the overlapping area if the target VLC APs are accessible. Once the HHO operation is complete, the original target VLC AP becomes the host VLC AP, and the new next VLC AP is evidently not accessible. In the continuous Markov chain, it is assumed that two events can never occur simultaneously [11,28].

The action space for the MDP is A={−1,0,1}, where 0 indicates the wireless access to RF APs, and 1 and −1 indicated the connection to the host VLC AP and the next VLC AP, respectively. Let p(i) denote the action taken at the beginning of the state *i*. A policy, denoted by p=(⋯,p(i),⋯), refers to the actions for all states. The handover decision is taken by the action p(i) and the current transmission mode *w*. For instance, assume both the host and the next VLC APs are available $\left(s,s^{\prime },h\right)=\left(\mathrm{On},\mathrm{On},2\right)$ and the VTM is connected to the host VLC AP (w=1) at the beginning. While the state is in transit to $\left(s,s^{\prime },h\right)=\left(\mathrm{Off},\mathrm{On},2\right)$, p(i) can be {1,0,−1}, where 1 means the VTM is waiting for the link recovery of the host VLC AP, and −1 and 0 represent the early HHO and VHO processes, respectively. Since early HHO can be executed for h=2 only, the action p(i) can be taken from {1,0,(−1)(h−1)} if it transits to a new state. We aim to find the optimal policy $p^{*}$ to maintain a high speed transmission rate with efficient handover cost.

Let ${\mathrm{Pr}}_{i\to i^{\prime }}\left\{\left(\mathrm{On},\;\mathrm{Off},\;1\right),p(i)\right\}$ denote the transition probability from state *i* to $i^{\prime }$ with action p(i). The transition probability for $i=\left(s,s^{\prime },h\right)=\left(\mathrm{On},\mathrm{Off},1\right)$ is given by

(2) $${\mathrm{Pr}}_{i\to i^{\prime }}\left\{\left(\mathrm{On},\;\mathrm{Off},\;1\right),p\left(i\right)\right\}=\left\{\begin{array}{cc} \frac{\lambda }{\lambda +\gamma_{1}+{\hat{\alpha }}_{12}+\mu_{\left(\mathrm{On},\;\mathrm{Off},1\right)}} & i^{\prime }=\left(\mathrm{On},\;\mathrm{Off},1,b+1,|p\left(i\right)|\right)\\ \frac{\gamma_{1}}{\lambda +\gamma_{1}+{\hat{\alpha }}_{12}+\mu_{\left(\mathrm{On},\;\mathrm{Off},1\right)}} & i^{\prime }=\left(\mathrm{Off},\mathrm{Off},1,b,|p\left(i\right)|\right)\\ \frac{{\hat{\alpha }}_{12}}{\lambda +\gamma_{1}+{\hat{\alpha }}_{12}+\mu_{\left(\mathrm{On},\;\mathrm{Off},1\right)}} & i^{\prime }=\left(\mathrm{On},\;\mathrm{On},2,b,|p\left(i\right)|\right)\\ \frac{\mu_{\left(\mathrm{On},\;\mathrm{Off},\;1\right)}}{\lambda +\gamma_{1}+{\hat{\alpha }}_{12}+\mu_{\left(\mathrm{On},\;\mathrm{Off},1\right)}} & i^{\prime }=\left(\mathrm{On},\;\mathrm{Off},1,b-1,|p\left(i\right)|\right) \end{array},\right.$$

where $\mu_{\left(\mathrm{On},\mathrm{Off},1\right)}=\left(1-|p\left(i\right)|\right)\mu_{0}+\frac{1}{2}p\left(i\right)\left(1+p\left(i\right)\right)\mu_{1}$. $\lambda +\gamma_{1}+{\hat{\alpha }}_{12}+\mu_{\left(\mathrm{On},\mathrm{Off},1\right)}$ is the sum of the transition rates, where ${\hat{\alpha }}_{12}=[1-\frac{1}{2}\left|p\left(i\right)|\left(1-p\left(i\right)\right)\right]\alpha_{12}$. ${\hat{\alpha }}_{12}$ is formulated for states $\left(s,s^{\prime },1\right)$ to avoid the action of accessing to the next VLC AP, since it is not available.

The transition probability for $\left(s,s^{\prime },h\right)=\left(\mathrm{On},\mathrm{Off},2\right)$ is given by

(3) $${\mathrm{Pr}}_{i\to i^{\prime }}\left\{\left(\mathrm{On},\;\mathrm{Off},\;2\right),p\left(i\right)\right\}=\left\{\begin{array}{cc} \frac{\lambda }{\lambda +\gamma_{1}+\beta_{-1}+\alpha_{21}+\mu_{\left(\mathrm{On},\mathrm{Off},2\right)}} & i^{\prime }=\left(\mathrm{On},\mathrm{Off},2,b+1,|p\left(i\right)|\right)\\ \frac{\gamma_{1}}{\lambda +\gamma_{1}+\beta_{-1}+\alpha_{21}+\mu_{\left(\mathrm{On},\mathrm{Off},2\right)}} & i^{\prime }=\left(textOff,\mathrm{Off},2,b,|p\left(i\right)|\right)\\ \frac{\beta_{-1}}{\lambda +\gamma_{1}+\beta_{-1}+\alpha_{21}+\mu_{\left(\mathrm{On},\mathrm{Off},2\right)}} & i^{\prime }=\left(\mathrm{On},\mathrm{On},2,b,|p\left(i\right)|\right)\\ \frac{\alpha_{21}}{\lambda +\gamma_{1}+\beta_{-1}+\alpha_{21}+\mu_{\left(\mathrm{On},\mathrm{Off},2\right)}} & i^{\prime }=\left(\mathrm{Off},\mathrm{Off},1,b,|p\left(i\right)|\right)\\ \frac{\mu_{\left(\mathrm{On},\;\mathrm{Off},\;2\right)}}{\lambda +\gamma_{1}+\beta_{-1}+\alpha_{21}+\mu_{\left(\mathrm{On},\;\mathrm{Off},\;2\right)}} & i^{\prime }=\left(\mathrm{On},\mathrm{Off},2,b-1,|p\left(i\right)|\right) \end{array},\right.$$

where $\mu_{\left(\mathrm{On},\mathrm{Off},2\right)}=\left(1-|p\left(i\right)|\right)\mu_{0}+\frac{1}{2}p\left(i\right)\left(1+p\left(i\right)\right)\mu_{1}$.

The transition probability for $\left(s,s^{\prime },h\right)=\left(\mathrm{On},\mathrm{On},2\right)$ is written as

(4) $${\mathrm{Pr}}_{i\to i^{\prime }}\left\{\left(\mathrm{On},\;\mathrm{On},\;2\right),p\left(i\right)\right\}=\left\{\begin{array}{cc} \frac{\lambda }{\lambda +\gamma_{1}+\gamma_{-1}+\alpha_{21}+\mu_{\left(\mathrm{On},\mathrm{On},2\right)}} & i^{\prime }=\left(\mathrm{On},\mathrm{On},2,b+1,|p\left(i\right)|\right)\\ \frac{\gamma_{1}}{\lambda +\gamma_{1}+\gamma_{-1}+\alpha_{21}+\mu_{\left(\mathrm{On},\mathrm{On},2\right)}} & i^{\prime }=\left(\mathrm{Off},\mathrm{On},2,b,|p\left(i\right)|\right)\\ \frac{\gamma_{-1}}{\lambda +\gamma_{1}+\gamma_{-1}+\alpha_{21}+\mu_{\left(\mathrm{On},\mathrm{On},2\right)}} & i^{\prime }=\left(\mathrm{On},\mathrm{Off},2,b,|p\left(i\right)|\right)\\ \frac{\alpha_{21}}{\lambda +\gamma_{1}+\gamma_{-1}+\alpha_{21}+\mu_{\left(\mathrm{On},\mathrm{On},2\right)}} & i^{\prime }=\left(\mathrm{On},\mathrm{Off},1,b,|p\left(i\right)|\right)\\ \frac{\mu_{\left(\mathrm{On},\mathrm{On},2\right)}}{\lambda +\gamma_{1}+\gamma_{-1}+\alpha_{21}+\mu_{\left(\mathrm{On},\mathrm{On},2\right)}} & i^{\prime }=\left(\mathrm{On},\mathrm{On},2,b-1,|p\left(i\right)|\right) \end{array},\right.$$

where $\mu_{\left(\mathrm{On},\mathrm{On},2\right)}=\left(1-|p\left(i\right)|\right)\mu_{0}+\left|p\left(i\right)\right|\mu_{1}$.

The transition rate for $\left(s,s^{\prime },h\right)=\left(\mathrm{Off},\mathrm{On},2\right)$ is formulated as

(5) $${\mathrm{Pr}}_{i\to i^{\prime }}\left\{\left(\mathrm{Off},\;\mathrm{On},\;2\right),p\left(i\right)\right\}=\left\{\begin{array}{cc} \frac{\lambda }{\lambda +\beta_{1}+\gamma_{-1}+\alpha_{21}+\mu_{\left(\mathrm{Off},\mathrm{On},2\right)}} & i^{\prime }=\left(\mathrm{Off},\mathrm{On},2,b+1,|p\left(i\right)|\right)\\ \frac{\beta_{1}}{\lambda +\beta_{1}+\gamma_{-1}+\alpha_{21}+\mu_{\left(\mathrm{Off},\mathrm{On},2\right)}} & i^{\prime }=\left(\mathrm{On},\mathrm{On},2,b,|p\left(i\right)|\right)\\ \frac{\gamma_{-1}}{\lambda +\beta_{1}+\gamma_{-1}+\alpha_{21}+\mu_{\left(\mathrm{Off},\mathrm{On},2\right)}} & i^{\prime }=\left(\mathrm{Off},\mathrm{Off},2,b,|p\left(i\right)|\right)\\ \frac{\alpha_{21}}{\lambda +\beta_{1}+\gamma_{-1}+\alpha_{21}+\mu_{\left(\mathrm{Off},\mathrm{On},2\right)}} & i^{\prime }=\left(\mathrm{On},\mathrm{Off},1,b,|p\left(i\right)|\right)\\ \frac{\mu_{\left(\mathrm{Off},\mathrm{On},2\right)}}{\lambda +\beta_{1}+\gamma_{-1}+\alpha_{21}+\mu_{\left(\mathrm{Off},\mathrm{On},2\right)}} & i^{\prime }=\left(\mathrm{Off},\mathrm{On},2,b-1,|p\left(i\right)|\right) \end{array},\right.$$

where $\mu_{\left(\mathrm{Off},\mathrm{On},2\right)}=\left(1-|p\left(i\right)|\right)\mu_{0}-\frac{1}{2}p\left(i\right)\left(1-p\left(i\right)\right)\mu_{1}$.

The transition rate for $\left(s,s^{\prime },h\right)=\left(\mathrm{Off},\mathrm{Off},2\right)$ is given by

(6) $${\mathrm{Pr}}_{i\to i^{\prime }}\left\{\left(\mathrm{Off},\;\mathrm{Off},\;2\right),p\left(i\right)\right\}=\left\{\begin{array}{cc} \frac{\lambda }{\lambda +\beta_{1}+\beta_{-1}+\alpha_{21}+\mu_{\left(\mathrm{Off},\mathrm{Off},2\right)}} & i^{\prime }=\left(\mathrm{Off},\mathrm{Off},2,b+1,|p\left(i\right)|\right)\\ \frac{\beta_{1}}{\lambda +\beta_{1}+\gamma_{-1}+\alpha_{21}+\mu_{\left(\mathrm{Off},\mathrm{Off},2\right)}} & i^{\prime }=\left(\mathrm{On},\mathrm{Off},2,b,|p\left(i\right)|\right)\\ \frac{\beta_{-1}}{\lambda +\beta_{1}+\gamma_{-1}+\alpha_{21}+\mu_{\left(\mathrm{Off},\mathrm{Off},2\right)}} & i^{\prime }=\left(\mathrm{Off},\mathrm{On},2,b,|p\left(i\right)|\right)\\ \frac{\alpha_{21}}{\lambda +\beta_{1}+\gamma_{-1}+\alpha_{21}+\mu_{\left(\mathrm{Off},\mathrm{Off},2\right)}} & i^{\prime }=\left(\mathrm{Off},\mathrm{Off},1,b,|p\left(i\right)|\right)\\ \frac{\mu_{\left(\mathrm{Off},\mathrm{Off},2\right)}}{\lambda +\beta_{1}+\beta_{-1}+\alpha_{21}+\mu_{\left(\mathrm{Off},\mathrm{Off},2\right)}} & i^{\prime }=\left(\mathrm{Off},\mathrm{Off},2,b-1,|p\left(i\right)|\right) \end{array},\right.$$

where $\mu_{\left(\mathrm{Off},\mathrm{Off},2\right)}=\left(1-|p\left(i\right)|\right)\mu_{0}$ because packets can only be transmitted by RF links for states $\left(s,s^{\prime }\right)=\left(\mathrm{Off},\mathrm{Off}\right)$. Similarly, the transition rate for $\left(s,s^{\prime },h\right)=\left(\mathrm{Off},\mathrm{Off},1\right)$ is written as

(7) $${\mathrm{Pr}}_{i\to i^{\prime }}\left\{\left(\mathrm{Off},\;\mathrm{Off},1\right),p\left(i\right)\right\}=\left\{\begin{array}{cc} \frac{\lambda }{\lambda +\beta_{1}+{\hat{\alpha }}_{12}+\mu_{\left(\mathrm{Off},\mathrm{Off},1\right)}} & i^{\prime }=\left(\mathrm{Off},\mathrm{Off},1,b+1,|p\left(i\right)|\right)\\ \frac{\beta_{1}}{\lambda +\beta_{1}+{\hat{\alpha }}_{12}+\mu_{\left(\mathrm{Off},\mathrm{Off},1\right)}} & i^{\prime }=\left(\mathrm{On},\mathrm{Off},1,b,|p\left(i\right)|\right)\\ \frac{{\hat{\alpha }}_{12}}{\lambda +\beta_{1}+{\hat{\alpha }}_{12}+\mu_{\left(\mathrm{Off},\mathrm{Off},1\right)}} & i^{\prime }=\left(\mathrm{Off},\mathrm{On},2,b,|p\left(i\right)|\right)\\ \frac{\mu_{\left(\mathrm{Off},\mathrm{Off},1\right)}}{\lambda +\beta_{1}+{\hat{\alpha }}_{12}+\mu_{\left(\mathrm{Off},\mathrm{Off},1\right)}} & i^{\prime }=\left(\mathrm{Off},\mathrm{Off},1,b-1,|p\left(i\right)|\right) \end{array},\right.$$

where $\mu_{\left(\mathrm{Off},\mathrm{Off},1\right)}=\left(1-|p\left(i\right)|\right)\mu_{0}$.

There is no packet departure for b=0 and no packet arrival for b=B, which leads to tiny differences in the transition rates for b=0 and b=B. For example, the transition rate of (Off,Off,2) for b=0 is

(8) $${\mathrm{Pr}}_{i\to i^{\prime }}\left\{\left(\mathrm{Off},\;\mathrm{Off},2\right),p\left(i\right)\right\}=\left\{\begin{array}{cc} \frac{\lambda }{\lambda +\beta_{1}+\beta_{-1}+\alpha_{21}} & i^{\prime }=\left(\mathrm{Off},\mathrm{Off},2,1,|p\left(i\right)|\right)\\ \frac{\beta_{1}}{\lambda +\beta_{1}+\gamma_{-1}+\alpha_{21}} & i^{\prime }=\left(\mathrm{On},\mathrm{Off},2,0,|p\left(i\right)|\right)\\ \frac{\beta_{-1}}{\lambda +\beta_{1}+\gamma_{-1}+\alpha_{21}} & i^{\prime }=\left(\mathrm{Off},\mathrm{On},2,0,|p\left(i\right)|\right)\\ \frac{\alpha_{21}}{\lambda +\beta_{1}+\gamma_{-1}+\alpha_{21}} & i^{\prime }=\left(\mathrm{Off},\mathrm{Off},1,0,|p\left(i\right)|\right) \end{array}.\right.$$

For b=B, the transition rate for (Off,Off,2) can be written as

(9) $${\mathrm{Pr}}_{i\to i^{\prime }}\left\{\left(\mathrm{Off},\;\mathrm{Off},\;2\right),p\left(i\right)\right\}=\left\{\begin{array}{cc} \frac{\beta_{1}}{\beta_{1}+\gamma_{-1}+\alpha_{21}+\mu_{\left(\mathrm{Off},\mathrm{Off},2\right)}} & i^{\prime }=\left(\mathrm{On},\mathrm{Off},2,B,|p\left(i\right)|\right)\\ \frac{\beta_{-1}}{\beta_{1}+\gamma_{-1}+\alpha_{21}+\mu_{\left(\mathrm{Off},\mathrm{Off},2\right)}} & i^{\prime }=\left(\mathrm{Off},\mathrm{On},2,B,|p\left(i\right)|\right)\\ \frac{\alpha_{21}}{\beta_{1}+\gamma_{-1}+\alpha_{21}+\mu_{\left(\mathrm{Off},\mathrm{Off},2\right)}} & i^{\prime }=\left(\mathrm{Off},\mathrm{Off},1,B,|p\left(i\right)|\right)\\ \frac{\mu_{\left(\mathrm{Off},\mathrm{Off},2\right)}}{\beta_{1}+\beta_{-1}+\alpha_{21}+\mu_{\left(\mathrm{Off},\mathrm{Off},2\right)}} & i^{\prime }=\left(\mathrm{Off},\mathrm{Off},2,B-1,|p\left(i\right)|\right) \end{array},\right.$$

Considering the early access option to the next VLC AP for all states of $\left(s,s^{\prime },2\right)$, the transition rate after action p(i) is written as

(10) $${\mathrm{Pr}}_{i\to i^{\prime }}\left\{\left(s,s^{\prime },2\right)\right\}=[1-\frac{1}{2}|p\left(i\right)|\left(1-p\left(i\right)\right)]{\mathrm{Pr}}_{i\to i^{\prime }}\left\{\left(s,s^{\prime },2\right),p\left(i\right)\right\}+\frac{1}{2}\left|p\left(i\right)\right|\left(1-p\left(i\right)\right){\mathrm{Pr}}_{i\to i^{\prime }}\left\{\left(s^{\prime },\mathrm{Off},\;1\right),p\left(i\right)\right\}$$

If p(i)=0or1, the transition rate ${\mathrm{Pr}}_{i\to i^{\prime }}\left\{\left(s,s^{\prime },2\right)\right\}$ equals ${\mathrm{Pr}}_{i\to i^{\prime }}\left\{\left(s,s^{\prime },2\right),p\left(i\right)\right\}$. If p(i)=−1, which means that early HHO is performed, the state transits to $\left(s^{\prime },\mathrm{Off},1\right)$. Therefore, ${\mathrm{Pr}}_{i\to i^{\prime }}\left\{\left(s^{\prime },\mathrm{Off},\;1\right),p\left(i\right)\right\}$ is the transition rate after early HHO.

The objective of the proposed handover decision problem is to minimize the weighted sum of the consumption for packet transmission and delay, as well as the switching cost. Therefore, the reward of this MDP is made up of two parts:

(11) $$g\left(i,p\left(i\right)\right)=\xi_{1}g_{1}\left(i,p\left(i\right)\right)+\xi_{2}g_{2}\left(i,p\left(i\right)\right),$$

where $g_{1}\left(i,p\left(i\right)\right)$ represents the sum of the energy cost for the packet transmission and latency, which is written as

(12) $$g_{1}\left(i,p\left(i\right)\right)=\left|p\left(i\right)\right|E_{V}+\left(1-p\left(i\right)\right)\left(1+p\left(i\right)\right)E_{R}+\zeta b,$$

where $E_{V}$ and $E_{R}$ are the energy consumed by the VLC and RF transmission, respectively. ζb is the delay cost incurred by the packets in the queue, with ζ the trade-off factor for a packet waiting to be processed. For analytical simplicity, the illumination energy of the LED lamps and the running cost of the base-stations are not included in $E_{V}$ and $E_{R}$. $g_{2}\left(i,p\left(i\right)\right)$ is the handover cost that takes the signaling cost of the link switching and the handover latency into consideration,

(13) $$g_{2}\left(i,p\left(i\right)\right)=\left|p\left(i\right)-w|(2-|p\left(i\right)-w|\right)\left(E_{V\;H\;O}+\theta \lambda \tau_{V\;H\;O}\right)+\frac{1}{2}|p\left(i\right)-w|(|p\left(i\right)-w\left|-1\right)\left(E_{H\;H\;O}+\theta \lambda \tau_{H\;H\;O}\right),$$

where the switching signaling cost for VHO and HHO are denoted by $E_{\mathrm{VHO}}$ and $E_{\mathrm{HHO}}$, respectively. The latency for VHO and HHO is represented by $\tau_{V\;H\;O}$ and $\tau_{H\;H\;O}$ so that the packets accumulated in the buffer during the VHO and HHO process are denoted by $\lambda \tau_{V\;H\;O}$ and $\lambda \tau_{H\;H\;O}$. θ is the latency coefficient decided by the delay requirement. $\xi_{1}$ and $\xi_{2}$ are the balance coefficients for $g_{1}\left(i,p\left(i\right)\right)$ and $g_{2}\left(i,p\left(i\right)\right)$. In simulations, $\xi_{1}>\xi_{2}$. This is because the QoS of the vehicular communication has to be guaranteed, although we aim to minimize the switching cost as well. Hence, the weight factor for the switching cost $\xi_{2}$ is smaller.

### 3.2. The Time Discretization

Uniformization is applied to transform the continuous time Markov chain into a discrete one, so that the formulated MDP is more analytically tractable.

Let $v_{i,p(i)}$ denote the total transition rate for state $i=(s,s^{\prime },h)$ with action p(i) (e.g., $v_{\left(\mathrm{On},\;\mathrm{Off},\;1),p(i\right)}=\lambda +\gamma_{1}+{\hat{\alpha }}_{12}+\mu_{\left(\mathrm{On},\mathrm{Off},1\right)}$ ) and $v_{m}$ denotes the uniform transition rate, which is the maximum of all the total transition rates, thus given by

(14) $$\begin{array}{c} v_{m}=\max_{i,p(i)}v_{i,p(i)} \end{array}$$

Define the discrete time transition probability for state $i=(s,s^{\prime },h)$ with action p(i) as ${\hat{\mathrm{Pr}}}_{i\to i^{\prime }}\left\{i,p\left(i\right)\right\}$, which can be derived by [26]

(15) $${\hat{\mathrm{Pr}}}_{i\to i^{\prime }}\left\{i,p\left(i\right)\right\}=\left\{\begin{array}{cc} \frac{v_{i,p(i)}}{v_{m}}{\hat{\mathrm{Pr}}}_{i\to i^{\prime }}\left\{i,p\left(i\right)\right\} & \mathrm{if}\;i\neq i^{\prime }\\ 1-\frac{v_{i,p(i)}}{v_{m}} & \mathrm{if}\;i=i^{\prime } \end{array}\right.$$

Then, the cost after uniformization, denoted by $\hat{g}\left(i,p\left(i\right)\right)$, is reformulated as

(16) $$\hat{g}\left(i,p\left(i\right)\right)=\frac{1}{\beta +v_{m}}g_{1}\left(i,p\left(i\right)\right)+g_{2}\left(i,p\left(i\right)\right),$$

where β is a parameter related to the discounted factor α as $\alpha =v_{m}/\left(\beta +v_{m}\right)$ [26]. α is the discounted factor of the proposed MDP, which is close to but smaller than 1.

### 3.3. Value Iteration Algorithm

Our goal is to minimize the expected value of the total cost by finding the optimal policy. A value function for state *i* with action p(i) is defined as the immediate cost of this state plus the expected sum of the cost of all future states [25],

(17) $$Q\left(i,p\left(i\right)\right)=\hat{g}\left(i,p\left(i\right)\right)+\alpha \sum_{i^{\prime }\in \Omega }{\hat{\mathrm{Pr}}}_{i\to i^{\prime }}\left\{i,p\left(i\right)\right\}V\left(i^{\prime }\right),$$

where α is the discount factor and $V(i^{\prime })$ is the value function of the next state. The value function for state *i* is defined as

(18) $$V\left(i\right)=\sum_{p(i)\in A}\mathrm{Pr}\left(p\left(i\right)|i\right)Q\left(i,p\left(i\right)\right),$$

where Pr(p(i)|i) is the probability of taking action p(i) in state *i*. If a deterministic policy is adopted, only one certain action is performed.

According to Bellman’s function, the optimal value function is defined as

(19)V(i)=minp(i)g^(i,p(i))+α∑i′∈ΩPr^i→i′{i,p(i)}V(i′)(20)=minp(i)Q(i,p(i))

Therefore, the dynamic programming of value iteration can be applied to find the optimal value function for each state as illustrated in Algorithm 1. With the optimal value function $V^{*}$, the optimal policy is derived by $p^{*}\left(i\right)=\min_{p(i)}\left\{Q^{*}\left(i,p\left(i\right)\right)\right\}$.
**Algorithm 1:** Value iteration algorithm**Input:** Ω; A; α; ${\hat{\mathrm{Pr}}}_{i\to i^{\prime }}\left\{i,p\left(i\right)\right\}$; $\hat{g}$; Convergence criterion parameter ϵ;**Output:**  Optimal Policy $p^{*}=\left(\cdots ,p^{*}\left(i\right),\cdots \right)$;**Initialize:** $V_{0}\left(i\right)=0$ for each i∈Ω; k=0; Δ=∞;**While** Δ>ϵ   **For** each i∈Ω      **For** each p(i)∈{1,0,(−1)(h−1)}          $Q_{k}\left(i,p\left(i\right)\right)=\hat{g}\left(i,p\left(i\right)\right)+\alpha \sum_{i^{\prime }\in \Omega }{\hat{\mathrm{Pr}}}_{i\to i^{\prime }}\left\{i,p\left(i\right)\right\}V_{k-1}\left(i^{\prime }\right)$;      **End For**       $p_{k}^{*}\left(i\right)=\min_{p(i)}\left\{Q_{k}\left(i,p\left(i\right)\right)\right\}$;      $V_{k}^{*}\left(i\right)=Q_{k}\left(i,p_{k}^{*}\left(i\right)\right)$;   **End For**   $\Delta =∥V_{k}-V_{k-1}∥$;**End While**

It is noted that the cost function $\hat{g}\left(i,g\left(i\right)\right)$ for each state is bounded. Hence, this iteration algorithm can be proved convergent [26]. The final handover decision for the considered vehicle VLC/RF communication system is made in terms of the optimal policy $p^{*}$ and the current transmission mode *w*.

With an optimal policy $p^{*}\left(i\right)$ and the state transition probability ${\hat{\mathrm{Pr}}}_{i\to i^{\prime }}\left\{i,p^{*}\left(i\right)\right\}$, the transition rate matrix can be written as P for all states $(s,s^{\prime },h,b,w)\in \Omega$. Let $\pi^{*}\left(s,s^{\prime },h,b,w\right)$ be the stationary probability for the state $\left(s,s^{\prime },h,b,w\right)$ and $\pi^{*}={\left(\cdots ,\pi^{*}\left(s,s^{\prime },h,b,w\right),\cdots \right)}^{T}$ the stationary probability vector. $\pi^{*}$ can be obtained by solving the following group of linear equations

(21) $$\begin{array}{cc} \pi^{*}P & =\pi^{*} \end{array}$$

(22) $$\begin{array}{cc} \pi^{*}1 & =1 \end{array}$$

where 1 is a column vector of ones.

Packet blocking occurs when a new packet arrives at states of b=B, which results in buffer-overflowing. Therefore, the sum probability of all states with the number of packets in the queue being *B* is the blocking probability of the packets, which can be calculated by

(23) $$P_{b}=\sum_{s,s^{\prime }\in S}\sum_{h\in H}\sum_{w\in W}\pi^{*}\left(s,s^{\prime },h,B,w\right).$$

When there are *B* packets waiting in the queue, the newly arrived packet will not be dropped if at least one packet can be delivered before the arrival time. Therefore, $P_{b}$ is the upper bound of the packet loss rate.

## 4. Simulation Results

In this section, the performance of the proposed hybrid handover scheme, labeled as O-HO, is compared with several benchmarks, listed as follows:

- Immediate Handover (I-HO): Handover is immediately performed when the current VLC link is interrupted. HHO is executed with high priority if the next VLC AP is accessible.
- Dwell Handover (D-HO): When blockage of the current VLC link is detected, the VTM waits for link recovery for a period of dwell time. If the VLC link is not recovered during waiting, handover is performed in the order of HHO and VHO. When the blocked VLC link is recovered, transmission with the host VLC AP continues.
- Immediate Vertical Handover (I-VHO): VHO is immediately performed once the current VLC link is interrupted.
- Dwell Vertical Handover (D-VHO): When blockage of the current VLC link is detected, the VTM waits for a period of dwell time. When the dwell time expires, VHO is performed if the VLC link has not been recovered. Otherwise, the transmission continues by the recovered VLC link to the host VLC AP.

In the benchmark handover schemes, the VTM is supposed to switch back to the VLC AP once the VLC link is recovered, because the VLC link is always preferable due to its high transmission rate. Thus, the dwell time helps avoid the potential ping-pong effect.

Four performance metrics were adopted to evaluate the handover schemes: *the packet loss rate* ρ, defined as the ratio of the number of the dropped packets to the total number of the arrived packets; *the average delay d*, defined as the average time a packet waits to be processed; *the average queue length l*, defined as the average number of packets waiting in the queue; and *the number of VHOs c*. The number of HHOs was not taken as a performance metric, because HHO would have been performed when the VTM moves and the signaling cost of HHO is much less than that of VHO.

Monte Carlo simulation was carried out over a period of continuous time. The simulation parameters are summarized in Table 1. The packet arrival and departures of RF and VLC links were modeled as Poisson processes with rates λ, $\mu_{0}$, and $\mu_{1}$, as stated before. The VLC–LoS link blockages of the host VLC AP and the next VLC AP were modeled as independently negatively exponentially distributed blocking events with duration parameters $\gamma_{1}$ and $\gamma_{-1}$. Similarly, the non-blocking duration for the LoS link of the host VLC AP and the next VLC AP were modeled with parameters $\beta_{1}$ and $\beta_{-1}$ [29]. The processes of VTM driving into the VLC APs’ overlapping areas and non-overlapping areas were also modeled as Poisson distributions with parameters $\alpha_{12}$ and $\alpha_{21}$, respectively. Since we focused on the switching cost of the handover process, the energy consumption of the VLC and RF transmission was assumed to be the same. The handover cost of HHO was assumed to be zero, because HHO would have been performed when driving. Finally, the performance metrics of the proposed hybrid handover scheme and the benchmarks were counted and calculated statistically.

**Figure 3:**
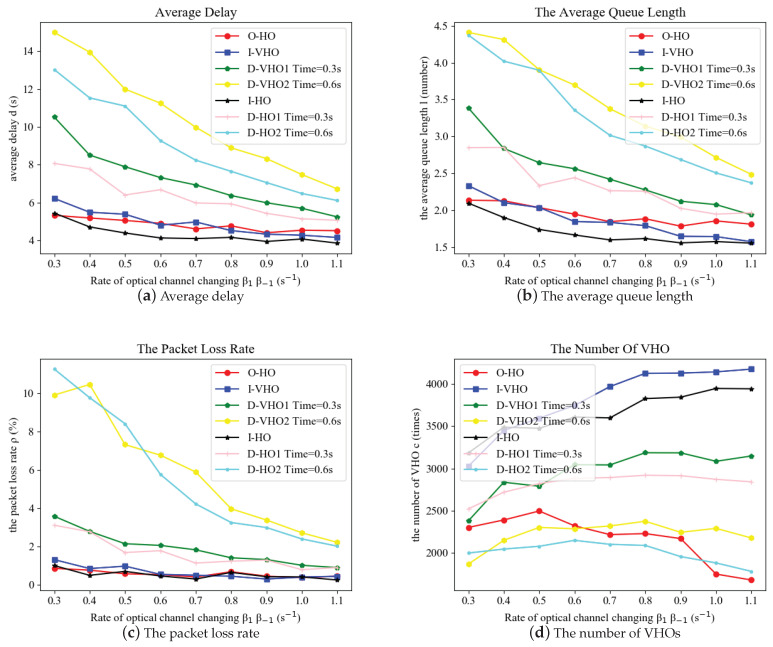
Performance comparison of the proposed optimal handover scheme and other benchmarks with respect to the recovery rate of VLC LoS links.

As shown in Figure 3, the four considered performance metrics were evaluated with respect to the link recovery rate of the VLC APs denoted by $\beta_{1}$ and $\beta_{-1}$, ranging from 0.3 to 1.1 ($s^{-1}$). The handover delays for HHO and VHO were set to $\tau_{H\;H\;O}=0.1\;s^{-1}$ and $\tau_{V\;H\;O}=0.3\;s^{-1}$ in the simulations. Larger values of $\beta_{1}$ and $\beta_{-1}$ indicate a shorter VLC link blockage duration and a greater chance of link recovery. Therefore, the average delay time, the average queue length, and the packet loss rate of all handover schemes decreased remarkably.

It can be observed that the I-HO, I-VHO, and the O-HO schemes significantly outperformed the others in terms of the average delay, the average queue length, and the packet loss rate. The average delay, the average queue length, and the packet loss rate for the I-HO, I-VHO, and the O-HO schemes were about a half of the corresponding performances for the Dwell handover scheme with a dwell time of 0.3 s and about a third for the Dwell time of 0.6 s. However, the VHO times of the I-HO and I-VHO schemes were much higher than the others, consuming too high a signaling cost of the heterogeneous networks, which might cause the ping-pong effect. The proposed optimal handover scheme, denoted by O-HO, achieved similar performance to the I-HO scheme, but performed a much lower number of VHOs (from two-thirds to lower than a half). Consequently, the O-HO scheme achieved a balance between the switching cost and the quality of service. In addition, the hybrid handover schemes (including O-HO, I-HO, and D-HO) obtained superior performance in respect of average delay time and average queue length compared to the counterpart VHO schemes (including I-VHO and D-VHO). This is because the proposed hybrid handover scheme introduced an early HHO decision that could avoid VHO signaling and provide continuous transmission links considering the driving state of the VTM.

**Figure 4:**
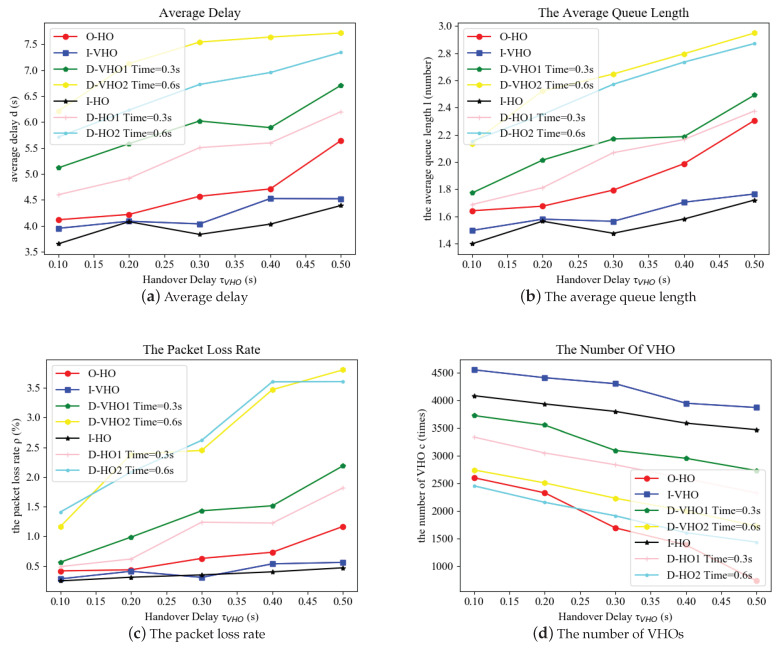
The performance comparison of the proposed optimal handover scheme and other benchmarks with respect to the handover delays.

Figure 4 illustrates the performances of all handover schemes versus the VHO delay $\tau_{V\;H\;O}$ with fixed VLC-link recovery rate $\beta_{1}=\beta_{-1}=1$. The VLC HHO delay between VLC APs was set to $\tau_{H\;H\;O}=0.1\;s$ and the VHO delay $\tau_{V\;H\;O}$ was increased from 0.1s to 0.5s. The increment in the gap between $\tau_{V\;H\;O}$ and $\tau_{H\;H\;O}$ degraded the performance of the average delay time, average queue length, and the packet loss rate. Because the cost function in (11) contained a positive weighted item of $\tau_{V\;H\;O}$, the value iteration algorithm tended to perform HHO or keep the transmission links unchanged so as to lower the total cost. As a result, the evaluated performance deteriorated but the VHO number was reduced.

According to Figure 4a–c, the performance of the I-HO and I-VHO was the best in terms of the average delay, the average queue length, and the packet loss rate, and it was least degraded by the increasing VHO delay, while the performance of the Dwell handover schemes with dwell times 0.3 s and 0.6 s was roughly 1.5 times and 2 times worse when the vertical handover delay reached its maximum. However, the I-HO and I-VHO schemes carried out the handover process without waiting, thus resulting in the highest VHO numbers. Moreover, the VHO numbers of the I-VHO scheme were higher than that of I-HO because early HHO was not considered as an alternative option. The proposed O-HO scheme achieved a performance slightly inferior to the I-HO, with a greatly reduced number of VHOs that was almost the same as the Dwell HO with a dwell time of 0.6 s. This is because the O-HO took into consideration the early HHO choice in the handover decision process.

## 5. Conclusions

In this paper, a hybrid handover scheme was put forward for vehicular VLC/RF communication networks. When a LoS link blockage happened during VLC signal transmission, the hybrid handover scheme aimed to make a decision about switching to the next VLC AP, to the RF AP, or waiting for link recovery, with full consideration of the state of the vehicle terminals. The handover decision problem was formulated as an MDP and the optimal policy was obtained using a dynamic programming method. The simulation results revealed that the proposed optimal handover policy achieved near-optimal performance with much lower VHO numbers, and thus was able to balance the QoS and the VHO signaling cost, and avoid the potential ping-pong effect in the heterogeneous systems. Furthermore, the performance of all hybrid handover strategies exceeded that of their VHO counterparts, benefiting from the newly introduced early HHO option.

## Figures and Tables

**Table 1:** Simulation parameters.

Parameter	Value
Packet arrival rate λ	2 Packets/s
Packet departure rate of VLC $\mu_{1}$	3.8 Packets/s
Packet departure rate of RF $\mu_{0}$	2.2 Packets/s
Changing rate of the host VLC AP from On → OFF $\gamma_{1}$	$0.4\;s^{-1}$
Changing rate of the next VLC AP from On → OFF $\gamma_{-1}$	$0.4\;s^{-1}$
Changing rate of the host VLC AP from OFF → On $\beta_{1}$	0.3–$1.1\;s^{-1}$
Changing rate of the host VLC AP from OFF → On $\beta_{-1}$	0.3–$1.1\;s^{-1}$
Changing rate from the overlapping area of VLC APs to the non-overlapping area	$\alpha_{21}=0.4\;s^{-1}$
Changing rate from the non-overlapping area of VLC APs to the overlapping area	$\alpha_{12}=0.2\;s^{-1}$
Energy consumption for transmissions by VLC links $E_{V\;L\;C}$	$5\times 10^{-4}$ kwh
Energy consumption for transmissions by RF links $E_{R\;F}$	$5\times 10^{-4}$ kwh
Handover delay for HHO $\tau_{H\;H\;O}$	0.1 s
Handover delay for VHO $\tau_{V\;H\;O}$	0.1–0.5 s
Signaling cost for VHO $E_{V\;H\;O}$	418 J
Signaling cost for HHO $E_{H\;H\;O}$	0 J
Buffer size *B*	10 Packets
Scaling parameter θ	1000 J/Packet
Trade-off Parameter ζ	500 J
Dwell time $t_{0}$	0.3 s, 0.6 s
Simulation Period	40,000 s
Learning rate α	0.99
Convergence criterion parameter ϵ	$10^{-5}$
Balancing coefficient $\xi_{1}$	1
Balancing coefficient $\xi_{2}$	0.75

## Data Availability

The data presented in this study are available on request from the corresponding author.

## References

[1] Eltokhey M.W., Khalighi M.A., Ghassemlooy Z., Jungnickel V. (2023). Handover-Aware Scheduling for Small-and Large-Scale VLC Networks. IEEE Trans. Netw. Serv. Manag..

[2] Mayahi M., Loscri V., Costanzo A. INVISIBLE: Enhanced Handover technique for Vehicular Visible Light Networks. Proceedings of the 2022 IEEE 95th Vehicular Technology Conference: (VTC2022-Spring).

[3] Hou J., O’Brien D. (2006). Vertical handover-decision-making algorithm using fuzzy logic for the integrated Radio-and-OW system. IEEE Trans. Wirel. Commun..

[4] Camporez H.A.F., Costa W.S., Silva J.A.L., Rocha H.R.O., Segatto M.E.V. Performance Evaluation of a Soft Handover Framework Applied to VLC Systems. Proceedings of the 2021 SBMO/IEEE MTT-S International Microwave and Optoelectronics Conference (IMOC).

[5] Dang Q.H., Yoo M. (2017). Handover Procedure and Algorithm in Vehicle to Infrastructure Visible Light Communication. IEEE Access.

[6] Jarchlo E.A., Gawłowicz P., Doroud H., Siessegger B., Jung M., Caire G., Zubow A., Ghassemlooy Z. A Flexible Transport Layer Protocol Architecture for Handover in a Vehicular VLC Network. Proceedings of the 2020 12th International Symposium on Communication Systems, Networks and Digital Signal Processing (CSNDSP).

[7] Demir M.S., Eldeeb H.B., Uysal M. (2020). CoMP-Based Dynamic Handover for Vehicular VLC Networks. IEEE Commun. Lett..

[8] Demir M.S., Miramirkhani F., Uysal M. Handover in VLC networks with coordinated multipoint transmission. Proceedings of the 2017 IEEE International Black Sea Conference on Communications and Networking (BlackSeaCom).

[9] Ozyurt A.B., Tinnirello I., Popoola W.O. Modelling of Multi-Tier Handover in LiFi Networks. Proceedings of the 2021 IEEE Global Communications Conference (GLOBECOM).

[10] Chen L., Li H. An MDP-based vertical handoff decision algorithm for heterogeneous wireless networks. Proceedings of the 2016 IEEE Wireless Communications and Networking Conference.

[11] Wang F., Wang Z., Qian C., Dai L., Yang Z. (2015). Efficient vertical handover scheme for heterogeneous VLC-RF systems. IEEE/OSA J. Opt. Commun. Netw..

[12] Guo D., Jin X., Deng J., Liu W., Jin M., Li S., Gong C., Xu Z. Load Balancing with Soft Handover for Indoor Hybrid VLC/WiFi Networks. Proceedings of the 2020 Asia Communications and Photonics Conference (ACP) and International Conference on Information Photonics and Optical Communications (IPOC).

[13] Huang S., Chuai G., Gao W. Two-Way Selection Handover Algorithm for Load Balancing in Hybrid VLC-RF Networks. Proceedings of the 2021 IEEE/CIC International Conference on Communications in China (ICCC).

[14] Shao S., Liu G., Khreishah A., Ayyash M., Elgala H., Little T.D.C., Rahaim M. (2020). Optimizing Handover Parameters by Q-Learning for Heterogeneous Radio-Optical Networks. IEEE Photonics J..

[15] Khoder R., Naja R., Mouawad N., Tojme S. Vertical Handover Network Selection Architecture for VLC Vehicular Platoon Driving Assistance. Proceedings of the 2020 IEEE 31st Annual International Symposium on Personal, Indoor and Mobile Radio Communications.

[16] Zang S., Bao W., Yeoh P.L., Vucetic B., Li Y. (2019). Managing Vertical Handovers in Millimeter Wave Heterogeneous Networks. IEEE Trans. Commun..

[17] Islam N., Kandeepan S., Chavez K.G., Scott J. A MDP-based Energy Efficient and Delay Aware Handover Algorithm. Proceedings of the 2019 13th International Conference on Signal Processing and Communication Systems (ICSPCS).

[18] Liu Q., Shi L., Sun L., Li J., Ding, M, Shu, F (2020). Path Planning for UAV-Mounted Mobile Edge Computing With Deep Reinforcement Learning. IEEE Trans. Veh. Technol..

[19] Okine A.A., Yun L., Nkurunziza P. An MDP-based Link Switching Scheme for WiFi-Infrared Heterogeneous Uplink Systems. Proceedings of the 2021 26th IEEE Asia-Pacific Conference on Communications (APCC).

[20] Khodmi A., Rejeb S.B., Nasser N., Choukair Z. MDP-Based Handover In Heterogeneous Ultra-Dense Networks. Proceedings of the 2021 International Conference on Information Networking (ICOIN).

[21] Wang L., Han D., Zhang M., Wang D., Zhang Z. (2021). Deep Reinforcement Learning-Based Adaptive Handover Mechanism for VLC in a Hybrid 6G Network Architecture. IEEE Access.

[22] Yan Z., Jaafar W., Selim B., Tabassum H. Multi-UAV Speed Control with Collision Avoidance and Handover-Aware Cell Association: DRL with Action Branching. Proceedings of the GLOBECOM 2023—2023 IEEE Global Communications Conference.

[23] Liu Q., Li X., Ji H., Zhang H. User Grouping-Based Beam Handover Scheme with Load-Balancing for LEO Satellite Networks. Proceedings of the GLOBECOM 2023—2023 IEEE Global Communications Conference.

[24] Ma C., Li J., Ding M., Yang H.H., Shu, F, Queck, T (2020). Q.S.; Poor, H.V. On Safeguarding Privacy and Security in the Framework of Federated Learning. IEEE Network..

[25] Sutton R., Barto A. (2020). Reinforcement Learning: An Introduction.

[26] Bertsekas D. (2007). Dynamic Programming and Optimal Control.

[27] Nguyen T., Chowdhury M.Z., Jang Y.M. Flexible Resource Allocation Scheme for Link Switching Support in Visible Light Communication Networks. Proceedings of the IEEE International Conference on ICT Convergence (ICTC).

[28] Bertsekas D., Tsitsiklis J. (2008). Introduction to Probability.

[29] Wang J., Venkatesha Prasad R., Niemegeers I. (2010). Solving the uncertainty of vertical handovers in multi-radio home networks. Comput. Commun..

